# Exploring presentations of sustainability by US synthetic biology companies

**DOI:** 10.1371/journal.pone.0257327

**Published:** 2021-09-17

**Authors:** James Karabin, Izaac Mansfield, Emma K. Frow

**Affiliations:** 1 School of Life Sciences, Arizona State University, Tempe, AZ, United States of America; 2 T. Denny Sanford School of Social and Family Dynamics, Arizona State University, Tempe, AZ, United States of America; 3 School for the Future of Innovation in Society, Arizona State University, Tempe, AZ, United States of America; 4 W.P. Carey School of Business, Arizona State University, Tempe, AZ, United States of America; 5 School of Biological & Health Systems Engineering, Arizona State University, Tempe, AZ, United States of America; University of Granada: Universidad de Granada, SPAIN

## Abstract

The field of synthetic biology is increasingly being positioned as a key driver of a more sustainable, bio-based economy, and has seen rapid industry growth over the past 15 years. In this paper we undertake an exploratory investigation of the relationship between sustainability and synthetic biology, identifying and analyzing sustainability-related language on the public websites of 24, US-based synthetic biology companies. We observe that sustainability is a visible part of the self-presentation of the nascent synthetic biology industry, explicitly mentioned by 18 of the 24 companies. The dominant framing of sustainability on these company websites emphasizes environmental gains and “free-market” approaches to sustainability, with little explicit mention of social dimensions of sustainability such as access, justice or intergenerational equity. Furthermore, the model of sustainability presented focuses on incremental transition towards environmental sustainability through direct substitution of products and processes using bioengineered alternatives (*n* = 16 companies), with no change in societal consumption or policy frameworks required in order to see sustainability gains. One-third of the companies analyzed (*n* = 8) mention “nature” on their websites, variously framing it as a resource to be managed or as a source of inspiration; whether the latter signals a potentially more complex relationship with nature than advanced free-market models of sustainability remains to be seen. As the synthetic biology industry begins to grow in size and visibility, we suggest this is an opportune time for the community to engage in explicit deliberation about its approach to sustainability.

## Introduction

Synthetic biology is a 21st-century approach to genetic engineering focused on developing infrastructure (tools, methods, platforms) and know-how for the systematic design and construction of genetic components, circuits, and organisms to carry out specific functions. It is being presented as enabling transformative advances across the biotechnology sector, including in food and agriculture [1, 2], drug and vaccine discovery and production [3, 4], material sciences [5, 6], and data storage [7]. Roles for synthetic biology are also being advanced for environmental challenges including climate change [8] and biodiversity conservation [9].

As the synthetic biology industry reports record levels of investment [10, 11] and its products are beginning to appear on the market [12], there is excitement regarding its prospects for contributing to the development of an increasingly bio-based economy. US policymakers define the bioeconomy in technological terms: “economic activity that is driven by research and innovation in the life sciences and biotechnology” [13]. Networks including SynBioBeta and industry groups such as the Bioceconomy Alliance are working to position synthetic biology at the heart of this bioeconomy [14]. A key ambition associated with the bioeconomy across many national bioeconomy strategies is achieving more environmentally conscious, sustainable growth and development through innovation using (renewable) biological resources [13, 15, 16].

There has so far been little scholarship examining the relationship between synthetic biology and sustainability [17–20]. This is complicated by the contested meaning of sustainability itself [21, 22]. Multiple, competing definitions of and approaches to sustainability exist. Almost all of them identify an environmental concern with preventing ecological depletion, while placing different emphases on how social and economic issues are interwoven with these environmental concerns [23]. In practice, public and media discourse around sustainability often focus narrowly on its environmental dimensions, in particular using language of environmentally friendly development and placing environmental and economic activities center-stage for improving societal well-being [24]. The role of technology is highly contested across different sustainability frameworks, ranging from technocentric approaches that embrace the use of technology to foster win-win-win gains across environmental, social and economic spheres, to more ecologically-grounded approaches that are highly skeptical of technology-based solutions [22].

In principle, there are several ways in which synthetic biology might contribute to more sustainable development, but sustainability should not be assumed as an inevitable outcome–there are simultaneously possibilities for the use of synthetic biology to exacerbate existing social inequalities and to cause environmental harm [25]. Some have also challenged the rhetoric of “abundance” that is often implicit when discussing the renewable nature of self-replicating organisms for biomanufacturing applications [26–28]. The contested meaning of sustainability means that both rhetorical and evidence-based claims of achieving sustainability through synthetic biology are open to challenge. Critically engaging with how sustainability is being presented can offer signals of how sustainability stands to be actualized in the synthetic biology industry. To this end, here we explore whether and how the nascent synthetic biology industry is positioning itself with respect to sustainability. To provide an empirical starting point, we conducted a pilot analysis of how sustainability is being presented on the websites of a sample of 24 US-based synthetic biology companies. How sustainability is being framed by this industry has implications for the material practice of sustainability, what it is that might be sustained, and the nature of any transition to sustainability.

## Methods

### Sample parameters

**Fig 1:**
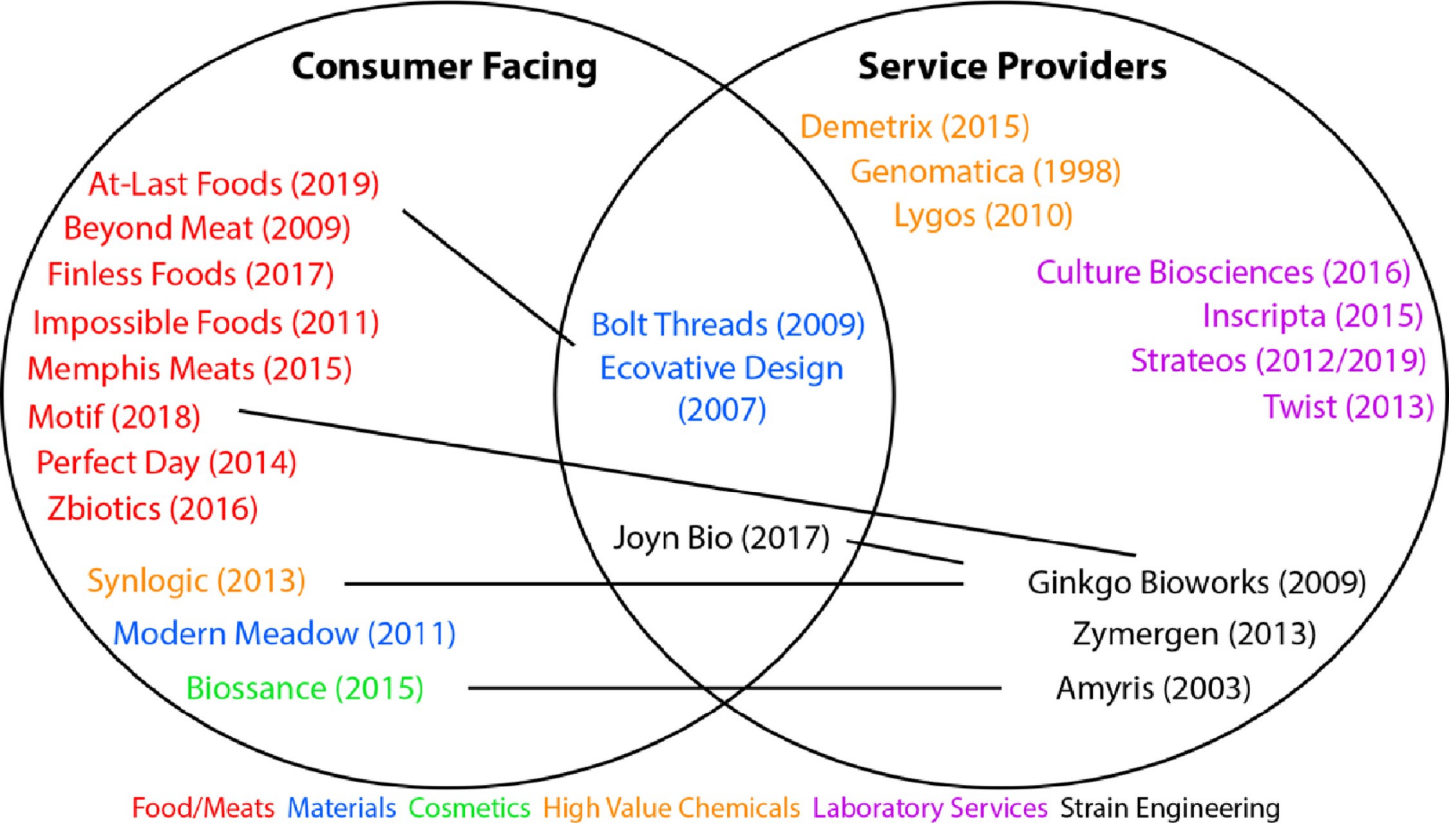
Overview of synthetic biology companies analyzed. Companies are categorized according to whether they are primarily consumer-facing companies or service providers, or have a hybrid model. Companies are also color-coded according to the focus of their key products / services. Year of company founding is indicated in parentheses. Solid lines between companies indicate spin-out companies and/or strategic investments by the older company.

For our pilot study we identified a sample of 24 US-based synthetic biology companies (Fig 1) that maintain public-facing websites. This sample represents approximately 25% of the (non-medical) US synthetic biology companies founded since the emergence of synthetic biology in the late 1990s. It is biased towards more mature companies that have pursued substantial venture capital and commercial investment (see for example the annual SynBioBeta investment reports), that have more detailed websites, and are actively supplying products to clients, with a smaller number of newer companies still primarily in development phases. Following [29], approximately half the companies in this sample occupy primarily “service provider” roles, and the other half are more “consumer-facing.” Service providers offer products or services (e.g. DNA synthesis, genetically engineered microorganism strains, or scale-up capabilities) directly to other companies or laboratories, while consumer-facing companies market products directly to public consumers. The sample was constructed to include companies using synthetic biology for a variety of applications (see Fig 1). Companies focusing exclusively on pharmaceuticals and therapeutics were excluded from analysis. We also focused primarily on companies doing at least some work directly with biological materials rather than those focused solely on equipment or software development [30].

### Data collection and analysis

Over a three-week period from mid-March to early April 2020, the websites for these 24 companies were read multiple times, and any language relating to sustainability was systematically extracted and categorized. The first read of a given company’s website involved straightforward cataloguing of all explicit mentions of the terms “sustainable” or “sustainability.” Any links to company-produced reports relating to sustainability were also documented.

To gain a deeper understanding of how synthetic biology companies position themselves with respect to sustainability, company websites were re-read to identify language that implicitly or explicitly related to different dimensions, definitions and approaches to sustainability. This was an iterative process of data collection and qualitative data coding [31] involving regular coordination among the researchers (JK, IM, EKF) to agree upon the scope and classification of material.

The researchers re-read each company’s website multiple times, each time attending to a different approach to or definition of sustainability, to identify which approach(es) to sustainability were visible in the company’s self-presentation. First, the commonly cited “three pillars” definition of sustainability [32] was used to identify and catalog which dimensions (environmental, social, economic) were present on a company’s website, and what particular aspects of each dimension were mentioned.

Second, mentions of the types of product(s) under development by each company were catalogued and categorized according to whether or not they represent a direct substitution of existing market products or processes. This category relates to literature on sustainability transitions, and was applied to understand whether sustainability through synthetic biology is presented as achievable through direct technological substitution or is framed as involving a more radical transformation process [33–35].

Third, all mentions of the term “nature” on the company websites were catalogued and characterized; this theme relates to distinctions in the literature between “free-market”, “thin” and “thick” versions of sustainability, which are differentiated primarily by their conception of nature and human-nature relationships [22, 36]. Free-market conceptions of sustainability typically frame nature as a resource, positioning humans as distinct from and in control of nature. Thin versions of sustainability ascribe some intrinsic value to nature, and aim to maintain some kind of dynamic equilibrium with the natural world while striving for “win-win” relationships between economic development and environmental protection. Thick versions of sustainability aim to sustain nature for its own sake, and are typically associated with deep ecology movements and prioritizing non-economic ways of measuring improvements in quality of life, well-being, and happiness [37].

Determination of the above categories occurred in an iterative manner involving deliberation among the researchers; that is, categories were derived through a process of reading the company website material and identifying possible points of intersection with existing debates around sustainability as themes by which to code and organize the raw data. Across multiple reads, the extracted material for each company’s website was organized using the following categories and codes:

- **Environmental sustainability**: references to (1) the environment, (2) the planet, (3) greenhouse gas emissions, (4) resource consumption.
- **Social sustainability**: Codes derived from the company websites included: references to (1) human health, (2) human well-being, (3) equity, (4) diversity, (5) accessibility. Owing to the complex and currently underdeveloped conception of social sustainability [37, 38], a very inclusive approach was taken to what was included within the scope of statements relating to social sustainability, including broad visions of the future with appeals to different aspects of culture, health, and well-being.
- **Economic sustainability**: mentions of (1) cost, (2) scale.
- Explicit references to “**nature**,” with the following codes: (1) nature as a resource to control, (2) nature as a resource to work with / learn from, (3) nature as a source of technical inspiration, (4) nature as a source of creative inspiration.

Finally, each sustainability-related statement was evaluated according to whether it referenced sustainability in a “substantive” or “unspecific” manner. “Substantive” statements had to mention a specific aspect of sustainability the company was working towards or a metric the company could use to evaluate its progress towards sustainability (even if no specific evidence or progress towards sustainability was provided). “Unspecific” statements referenced sustainability without providing a measurable indication of how sustainability might be understood or evaluated.

Data collection was limited to text presented on the public websites of synthetic biology companies; external news coverage or reports written about a given company were excluded, as they are not directly controlled by the company. All webpages containing relevant data were captured and archived using screen capture software, and sustainability-related text was extracted and coded in an Excel spreadsheet. Each researcher (JK, IM, EKF) undertook independent coding of all the material, and any instances of inter-coder variation were discussed among all three authors until consensus was achieved regarding the classification of each individual statement.

## Results

### Sustainability is a visible part of the synthetic biology industry

**Fig 2:**
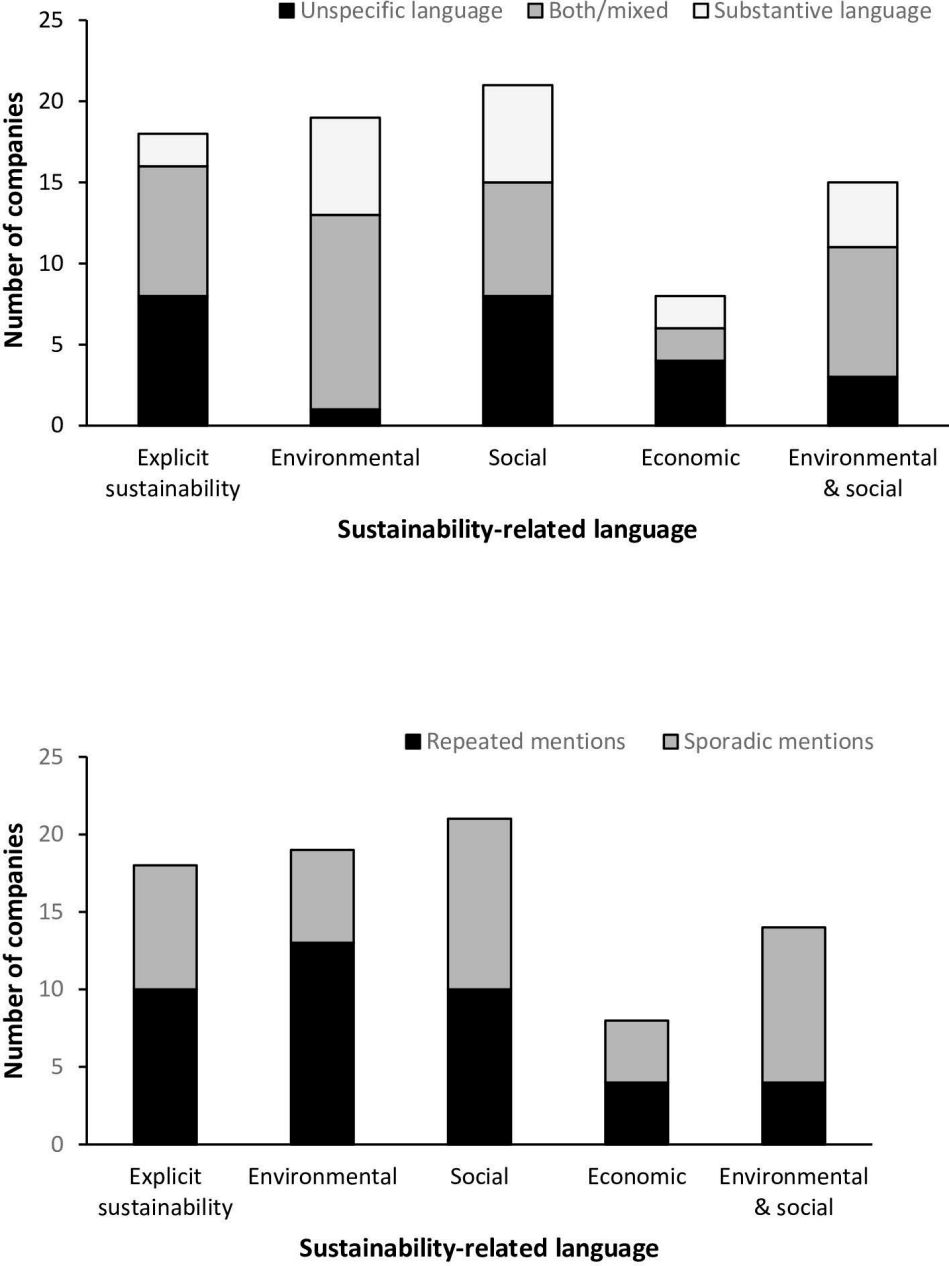
Presence of sustainability language on company websites. The presence of sustainability-related language was tabulated for each of 24 synthetic biology companies. The language for each company was characterized according to **(a)** the dimensions of sustainability invoked, and whether the sustainability-related language was unspecific, more substantive, or contained a mix of unspecific and more substantive claims, and **(b)** whether sustainability-related language was repeatedly invoked, or only sporadically mentioned (fewer than 3 times across a given company’s website).

Of the 24 companies examined, 18 explicitly used the terms “sustainable” or “sustainability” (Fig 2A), and a total of 22 used sustainability-related language. Only two companies lacked any language related to sustainability. Examining the dimensions of sustainability invoked by companies, 19 out of 24 used language relating to environmental sustainability, and 21 invoked aspects of social sustainability. Economic language was less often used, observed in 8 out of the 24 companies.

We further evaluated the sustainability language used by individual companies with respect to their frequency of use (Fig 2A) and the substantiveness of sustainability-related statements (Fig 2B and Table 1). We observe heterogeneity across the companies sampled with respect to the level of detail provided in their sustainability-related language. Only a small number consistently discussed sustainability in substantive ways, by identifying particular metrics or aspects of sustainability they were working towards. Five companies provided some formal documentation or discussion of sustainability on their websites. Examples include life-cycle analysis reports (Beyond Meat), impact reports (Impossible Foods), corporate social responsibility reports (Genomatica), as well as less traditional materials, such as the Creative Resident blog posts and *Grow* magazine published by Ginkgo Bioworks. Other companies, such as Amyris and Biossance, explicitly mentioned sourcing input materials that have been formally certified as sustainable (e.g. sugarcane). In contrast, most companies used sustainability-related language without offering specific evidence or metrics to qualify their claims; this language was often utopic, painting positive visions of the future and emphasizing win-win scenarios for consumers and the environment but without specifying particular sustainability outcomes or actions. Table 1 contains representative examples of substantive and unspecific sustainability language.

**Table 1:** Representative examples of unspecific and more substantive sustainability-related language on company websites.

Sustainability dimension	Unspecific language	More substantive language
Explicit mentions of sustainability	“Safe for the planet, sustainable and cruelty-free.” (Biossance)	“Every year we publish an Impact Report, documenting our progress toward a more sustainable food system.” (Impossible Foods)
	“We’re exploring and expanding the metabolic map, finding new pathways to sustainable products.” (Ginkgo Bioworks)	“Silk proteins are inherently biodegradable and can be produced in a sustainable, closed-loop process.” (Bolt Threads)
Environmental	“We are on a mission to make a measurable difference, for both the environment and quality of life.” (Atlast Food Co.)	“We aim to make meat better for the planet and all its inhabitants, while using significantly less land and water. At scale, our process will create less waste while dramatically reducing greenhouse gas emissions.” (Memphis Meats)
	“Making the world a kinder, greener place.” (Perfect Day)	“Cruelty-free, biodegradable, latex-free, vegan, petrol-free, eco-friendly” (Ecovative Design)
Economic	“Our target products are the specialty chemicals of today that can grow into the commodity chemicals of tomorrow.” (Lygos)	“For the last 75-years malonic acid has been produced from cyanide and chloroacetic acid, two costly and environmentally hazardous chemicals. The petrochemical production process has restricted market growth. Lygos’ technology uses sugar and water, opening the door to new applications.” (Lygos)
	“Our solution delivers products to market faster, cheaper, green, and simply better than ever before possible.” (Zymergen)	
Social	“This project illustrates how we can bring together innovative technologies, based on biology, to protect and benefit children worldwide” (Twist Bioscience)	“Our technology incorporates practices for ethical genome engineering. Contributions that power progress are shared. And every member of our team is valued for their unique perspective and contributions.” (Inscripta)
	“Happy, healthy lives” (Demetrix)	“Genetic engineering has given us incredible things: life-saving medicines, stress-resistant and more nutritious crops, production methods that use less resources and don’t require the killing of animals.” (Zbiotics)
Environmental *and* Social	“Our groundbreaking production method has a host of benefits—for our oceans, for the planet, and for you.” (Finless Foods)	“. . .working toward a world where everyone has access to healthy, delectable seafood, without the environmental devastation or the health hazards of traditional fishing and aquatic farming.” (Finless Foods)
	“Better for the people and better for the planet.” (Memphis Meats)	“Pesticide runoff, water-intensive crops, and petrochemical-based fertilizers all take a toll on the health of our people and planet. Healthier and sustainable solutions are not nice-to-haves, they’re must-haves for the future of food.” (Zymergen)

Examples are provided for each dimension of sustainability investigated on the sampled companies’ websites. Note that even the”more substantive” examples might not provide evidence of sustainability, but they do typically mention an action or a metric by which sustainability might be evaluated.

#### Environmental sustainability

Language relating to environmental dimensions of sustainability was visible on the websites of 19 out of the 24 companies. This category included any language that explicitly or implicitly referred to environmental well-being, either invoking specific markers of environmental health or making less specific claims about broad environmental benefit. Thirteen companies invoked environmental language multiple times across their websites, with 6 consistently using environmental language in substantive and detailed ways (Fig 2A and 2B; Table 1).

Among the 19 companies that used environmental language, consumer-facing companies were more likely to use unspecific language than providing concrete examples of their environmental sustainability aspirations (Fig 2B; Table 1). In contrast, service provider companies explicitly mentioned sustainability more frequently and made more substantive claims about environmental benefits.

#### Social sustainability

Language relating to social dimensions of sustainability was used by 21 of the 24 company websites examined. Of these, 11 companies invoked social language sporadically while 10 made more than three statements relating to social dimensions of sustainability. Regarding the nature of the statements, 8 companies used almost exclusively unspecific language, often relating to undefined “better” futures. A mix of unspecific and more substantive language was used by 7 companies, and 6 made consistent, substantive statements invoking social dimensions of sustainability (Fig 2A and 2B; examples in Table 1).

Recurring examples of the social language identified included broad appeals to health (“better for you”), mentions of “happiness”, discussion of specific values (e.g., veganism, animal cruelty), and diversity practices within the company (a theme that relates primarily to internal company practices). Across the companies studied, intergenerational dimensions of social sustainability were largely absent, as were explicit mentions of access and equity. We also identify that much of the social language used on company websites was accompanied with environmental language, invoking win-win progress towards sustainable development (*n* = 15) (see Table 1).

#### Economic sustainability

Economic dimensions of sustainability were least frequently represented, found on the websites of only 8 of the 24 companies studied; 4 of these companies mentioned economic matters multiple times across their websites, while the other 4 used economic language only once or twice (Fig 2A). Only 2 companies used substantive language relating to economic aspects of sustainability (Fig 2B; Table 1). Instances of economic language were typically present on the websites of service-provider companies, often describing the advantages to other companies of purchasing their products or services; they were rarely found on the websites of consumer-facing synthetic biology companies.

### The dominance of ‘free-market’ framings of sustainability

Statements relating to sustainability were analyzed with the aim of broadly understanding how synthetic biology companies are positioning themselves with respect to different frameworks and paradigms within sustainability discourse (see Methods). We identify that free-market framings of sustainability are prevalent among the company websites sampled.

#### Achieving sustainability through resource management and product substitution

Of the 22 companies that used sustainability-related language on their websites, 16 framed sustainability through the lens of directly substituting or replacing existing products and processes. Examples of substitutive language are shown in Table 2. All but one of these companies (*n* = 15) framed these substitutions as direct, one-to-one replacements of existing products, using synthetic biology to produce an equivalent substance to something currently in use (e.g. a specific food, chemical, material).

**Table 2:** Substitutive language used on company websites.

Company	Example	Alternative to
Impossible Foods	“We’re making meat from plants so that we never have to use animals again. That way, we can eat all the meat we want, for as long as we want. And save the best planet in the known universe.”	Animal meat
Finless Foods	“by bypassing the ocean and fish farming, we produce the same fish–but without the mercury, plastic and other environmental contaminants”	Fish
Perfect Day	“Flora-made dairy means dairy produced sustainably using less water, energy, greenhouse gas emissions and land.”	Dairy
Biossance	“our squalene saves 2 million sharks every year”	Animals (for cosmetic products)
Demetrix	“our highly specialized production system yield even the rarest ingredients with less impact on the planet than traditional ways of harvesting nature’s gifts”	Rare natural ingredients
Bolt Threads	“microsilkTM can be produced with less environmental impact than traditional textile manufacturing”	Textiles
Modern Meadow	“We are working toward commercial biofabricated materials that will be animal-free with a lighter footprint on the planet.”	Materials
Ecovative	“MycoFlexTM sustainable foam has superior properties compared with traditional petroleum-based materials”	Materials (from petrochemicals)
Genomatica	“we develop commercial biobased processes to make widely-used chemicals that enable better, more sustainable everyday products”	Chemical industry
Ginkgo Bioworks	“Cultured ingredients offer a more accessible and sustainable way to produce an array of important products across industries.”	Products across multiple industries
Ginkgo Bioworks	“organism engineers at Ginkgo learn from nature to develop new organisms that replace technology with biology	Technology

Representative examples are provided for different types of product or technology substitutions under development.

One company, Joyn Bio, stated the aim to “reduce agriculture’s reliance on synthetic nitrogen fertilizer,” but sought to do so through engineering microbes rather than by creating a biofertilizer replacement. The remaining 7 companies did not frame their innovations as directly substituting for existing processes or products; these companies fell primarily into the service-provider category (*n* = 5) rather than companies producing consumer-facing products.

For companies seeking to produce direct substitutions for existing products, the gains achieved through these substitutions were largely cast in environmental terms (e.g., resulting in changing land or water use patterns, reductions in greenhouse gas emissions), or just broadly framed as sustainability gains. Social benefits (e.g., benefits to health, improving accessibility) were referenced less often. Overall, the predominant framing was one of resource management for sustainability gains, consistent with win-win sustainability solutions (environmental protection alongside economic growth).

#### Relationships with nature

The position of humans with respect to nature underpins many of the differences across approaches to sustainability [22]. Different relationships with nature were visible in the data collected; of the 24 companies analyzed, 8 mentioned the word “nature” on their websites. Within this group, no clear, straightforward representation of nature emerged: the framing of statements regarding nature took different forms, sometimes within a single company and also across companies. We grouped these statements into 4 major categories, summarized with examples in Table 3.

**Table 3:** References to nature on company websites.

Relationship with Nature	Example	Company
Nature as a resource–Controlling / exploiting nature	“We take nature’s unpredictability out of the equation.”	Demetrix
	“We seek to transform the material world by unlocking the power of nature to inspire design for a healthier planet”	Modern Meadow
	“Unlocking nature’s superpowers”	Zymergen
Nature as a resource–Working with / learning from nature	“By working with nature, intelligently, we help our partners access the ingredients of life.”	Demetrix
	“Organism engineers at Ginkgo learn from nature to develop new organisms that replace technology with biology”	Ginkgo Bioworks
Nature as inspiration–Technical inspiration	“Nature offers superior solutions no matter the category.”	Zymergen
	“Biology is the most advanced manufacturing technology on the planet. Self-assembling, self-replicating, and self-repairing, biology builds renewably.”	Ginkgo Bioworks
	“Synthetic biology is a reimagining tool, allowing scientists to apply insights from nature to understand and design new biological systems, genetic circuits and molecular components to improve the world around us”	Synlogic Therapeutics
Nature as inspiration–Creative inspiration	“Inspired by nature and built by biology, we build better products in better ways for business, people, and the planet”	Zymergen
	“Taking nature as our inspiration we invent and scale advanced, credible materials that put us on a path towards a more sustainable future”	Bolt Threads

Representative examples are provided to illustrate different descriptions of the role of nature in a company’s work.

## Discussion

We set out to better understand how US synthetic biology companies are publicly signaling their intentions and positioning themselves with respect to sustainability. Overall, our findings show prevalent and heterogeneous use of sustainability-related language by these companies–through explicit use of the term, and by appealing to various facets (environmental, social, economic) of sustainability. This suggests that sustainability is a key aspect of how synthetic biology companies are presenting their value propositions within the biotechnology sector.

Below, we draw on these findings to offer two, somewhat divergent, interpretations of the current position of sustainability within the synthetic biology industry. This ambiguity is reflective of sustainability discourse more broadly [39], and should not necessarily be interpreted negatively. Rather, it draws attention to the opportunity (and arguably the need) for a more explicit and intentional discussion of sustainability within the synthetic biology industry, particularly as it faces growing market pressure to be commercially viable [40] and respond to broader imperatives around climate change and social responsibility.

### Free-market sustainability: Business as usual

According to the three-pillars definition of sustainability, no one pillar (social, environmental, economic) is presented as more foundational or essential than any other, but the emphasis among them may vary across specific contexts. We find that mentions of sustainability on synthetic biology company websites appeal most concretely to environmental dimensions of sustainability. Economic aspects of sustainability are infrequently mentioned. Mentions of social sustainability tend to focus broadly on desirable (“happy”, “healthy”) futures and rarely raise issues of justice or equity within or across generations; they are most specific when referring to internal company practices around diversity. While sustainability is thus a visible part of the messaging of the synthetic biology industry, the scope of what is included under the umbrella of sustainability thus far remains fairly limited, and is consistent with broader tendencies in public and marketing discourse to equate sustainability with environmentalism [24, 41, 42].

We identify that the predominant model of sustainability presented on synthetic biology company websites is one of technological “drop-ins” or substitutions that are largely invisible to the end consumer. This reflects an approach to sustainability grounded in the “technological fix,” largely dissociated from models of broader social or political change [43]. In the presentation of sustainability most visible on synthetic biology company websites, there is no need for society to decrease consumption in order to see sustainability gains. This presentation is most closely associated with dominant, “free-market” approaches to sustainability–which celebrate technological innovation–and stands in opposition to “thicker” views of sustainability that view overconsumption as a major contributing factor to unsustainability [22]. In this respect, synthetic biology companies might be described as supporting incremental transitions towards sustainability rather than fostering more fundamental transformation. We do not suggest this is necessarily negative, but it might be seen to fall short for a field that makes broad claims about its “revolutionary” potential [44, 45].

Furthermore, sustainability is often presented as something these companies (or their products) simply *are*, or something that will be straightforwardly achieved by these companies (or their products). Largely absent are explicit descriptions of specific actions a company is taking towards sustainability in the *present*. Through the current framing on company websites, sustainability remains something that will emerge in the future, rather than something that requires intentional efforts, imagination, and urgent collective action to be undertaken in the present. It allows technological advances to be the solution to sustainability, and in this way elides a need to grapple with more complex, sociopolitical dimensions of sustainability [17].

### Prospects of a “thicker” relationship with sustainability

This paper does not set out to evaluate whether synthetic biology companies are or are not actually achieving sustainability gains; we focus here on how they present their relationship to sustainability to a public audience. We acknowledge that surveying the outward-facing websites of synthetic biology companies cannot provide a comprehensive view of what companies might be doing in practice with respect to sustainability. But it does contribute to debates over what qualifies as sustainable practice, and offers public signals that can be critically assessed. Our findings show that about half the companies examined use at least some substantive language with respect to sustainability, most often pointing to specific types of environmental gains. It is also clear that some synthetic biology firms are devoting internal resources to detailing the nature and scope of sustainability gains made possible through their work (e.g. [20]), and about 20% of the companies examined here have publicly accessible reports or plans on their websites containing more detailed accounts of sustainability-oriented metrics and actions.

About one-third of the companies we examined seem to be signaling a different, potentially more nuanced relationship with nature than standard free-market models of sustainability typically assume. In particular, the idea of nature as a source of inspiration (rather than simply a physical resource or commodity) is visible on some company websites. At present, this framing remains ambiguous: while it might be less clearly “extractive” of nature, it does continue to position nature as distinct from humans (and thus does not reflect alignment with “thick” approaches to sustainability, see Methods). The line between inspirational and instrumental is also not clear-cut. While suggesting an approach to nature that goes beyond commodity management, it could still potentially be interpreted as somewhat extractive (i.e., the value of nature is in its utility as inspiration for product development, rather than valuing nature for its own sake). Furthermore, the idea of inspiration serves to link nature with human intention and ingenuity–a framing that is often used in technology-driven, free-market framings of sustainability as a way to decouple economic growth from environmental scarcity [17]. So while there are intriguing signs of a more complex relationship with nature emerging on the websites of US synthetic biology companies, there is ambiguity–both within the websites of individual companies, and across the industry as a whole–as to whether this is indicative of a foundationally different orientation to sustainability taking shape within the field of synthetic biology.

References to improvements in quality of life through use of terms like “better” and “happier” on company websites might also be seen to gesture towards thicker understandings of sustainability, which typically advocate for non-economic ways of understanding progress towards sustainability. However, in the current study such terms were rarely accompanied by any specificity in what kinds of social improvements might be seen or how the company proposed to document social sustainability gains, suggesting that these terms might currently be used more for marketing purposes than for signaling specific intentions regarding sustainability.

There are hints of broader, collaborative networks taking shape within the emergent synthetic biology ecosystem, typically involving artists, social scientists and humanists. For example, Zymergen has pursued sustainability-related research collaborations with social scientists [20]. The Creative Residency program and in-house magazine at Ginkgo Bioworks [46] are two venues creating space for interdisciplinary and critical discussions around sustainability, particularly in its social and cultural dimensions. For example, the 2019 Creative Resident focused on creating a world without waste, as a commentary on the extractive capitalist system within which synthetic biology operates [47]. Vos suggests that broadening out stakeholder participation is key to developing thicker versions of sustainability [22]. Environmental justice social movements, for example, have long worked on combining goals of environmentalism and social equity [35]. While deeper engagement might be seen as a daunting task for biotech companies [20], the field of synthetic biology is in principle well-positioned to build networks and foster broader societal dialogue around sustainability goals and how to achieve them, with its longstanding commitment to interdisciplinary collaboration, “human practices” [48] and “responsible innovation” efforts [49, 50].

## Conclusion

Sustainability remains an ambiguous and contested concept, including in the synthetic biology industry, which seems to be adopting sustainability as a key element of its value proposition. While it is natural that the heterogeneity in public sustainability discourse broadly is also reflected within this nascent industry, we suggest the synthetic biology community can play a leadership role in grappling explicitly with the particular characterizations of sustainability it wishes to emphasize, and the actions it will adopt in pursuit of these goals. Currently, the public presentation of sustainability by the US synthetic biology industry is aligned with broader industry trends towards environmental sustainability and free-market approaches to sustainability gains. There is little attention focused on social and equity dimensions of sustainability, and sustainability achievements are framed in terms of incremental substitutions towards an ill-defined “better” world.

With growing policy discussion and interest in positioning synthetic biology at the heart of a new bioeconomy, there is a risk that these currently limited sustainability discussions become subsumed under economic concerns around growth and competitiveness, becoming an assumed by-product rather than an explicit goal of this industry [51]. As the synthetic biology industry grows in size and visibility, how it chooses to publicly frame and discuss sustainability is important both to the future of this industry and to how sustainability is imagined and practiced more broadly. Whether the processes and products of synthetic biology will promote a step-wise, incremental transition in the material underpinnings of our economy, or generate and contribute to more radical, systemic transformation remains to be seen [35]. This is an opportune time to openly reflect on and debate the possibilities of what the industry is trying to sustain, for whom, and how to take concrete action in the present to support longer-term goals of sustainability.

## Supporting information

S1 Table Categorized statements from US synthetic biology company websites. This table contains all the statements extracted by the researchers from the websites of the 24 companies examined for this study, categorized according to whether they (1) explicitly invoke sustainability, refer to (2) environmental, (3) social, or (4) economic dimensions of sustainability, (5) explicitly mention “nature” or (6) are linked to a report or publication on the website containing sustainability-related material. Individual statements that fit into multiple categories are included in each of the relevant categories in a given row of the table.
(XLSX)
